# Effect of High-Flow Nasal Cannula Oxygen Therapy on Hypoxemia in Patients After Esophagectomy

**DOI:** 10.1155/carj/4691604

**Published:** 2025-02-20

**Authors:** Yumei Shen, Yi Xu, Fanglan Xu, Xiaofan Wang, Shanzhou Duan, Yongbing Chen

**Affiliations:** ^1^Department of Operating Room, The Second Affiliated Hospital of Soochow University, Suzhou 215004, China; ^2^Department of Thoracic Surgery, The Second Affiliated Hospital of Soochow University, Suzhou 215004, China

**Keywords:** conventional oxygen therapy, esophageal cancer, high-flow nasal cannula, hypoxemia, postoperative complications, respiratory support

## Abstract

**Background:** Patients with esophageal cancer (EC) who have undergone esophagectomy are at risk of developing hypoxemia and encountering postoperative complications. It is essential to ascertain whether the high-flow nasal cannula (HFNC) therapy offers superior clinical efficacy compared to conventional oxygen therapy (COT).

**Methods:** Clinical data from 80 patients who experienced hypoxemia subsequent to radical esophagectomy were retrospectively collected at our institution spanning January 2020 to December 2022. The whole cohort was divided into two groups: the HFNC group and the COT group. Following oxygen administration, we evaluated the variations in arterial blood gas parameters and infection indices within each group, in addition to scrutinizing the occurrence of postoperative pulmonary complications.

**Results:** The HFNC group was associated with a better oxygenation index (*F*_group_=41.779, *p* < 0.001) and partial pressure of carbon dioxide (*F*_group_=16.760, *p* < 0.001) compared with the COT group. Moreover, there were statistically significant differences in the reduction of C-reactive protein (*F*_group_ = 17.603, *p* < 0.001) and neutrophil count (*F*_group_ = 4.395, *p*=0.039) in the HFNC group compared with the COT group after 3 days of oxygen therapy. Notably, patients treated with HFNC exhibited a markedly reduced risk of developing postoperative complications, especially pneumonia (*p*=0.039).

**Conclusion:** HFNC outperformed COT in enhancing oxygenation and reducing carbon dioxide levels and infection indices among patients with hypoxemia after radical resection of EC and also lowered the risk of postoperative pneumonia.

## 1. Introduction

Esophageal cancer (EC) is one of the most common malignancies of the gastrointestinal tract worldwide. Its incidence rate ranks are among the top 7 worldwide, and its mortality rate is among the top 6 [1]. Squamous cell carcinoma and adenocarcinoma predominantly characterize malignant tumors of the esophagus, with a variety of other subtypes present. Variations in the distribution of these histologic subtypes occur regionally, corresponding to regional disparities in incidence and mortality rates [2]. Currently, esophagectomy (ESO) remains the mainstay of treatment for resectable EC. Yet, due to the complexity of the technique, the postoperative complication rate is as high as 59%, with a 90-day mortality rate of 4.5% [3]. Among these, postoperative pulmonary infection stands out as particularly prevalent, directly contributing to increased mortality, extended hospital stays, decreased quality of life, and escalated hospital costs [3–5].

Meanwhile, respiratory support methods (e.g., high-flow nasal cannula [HFNC], continuous positive airway pressure ventilation, and continuous mechanical ventilation) were increasingly employed to mitigate or address pulmonary-related postoperative complications [6]. HFNC, capable of supplying a continuous flow of up to 60 L/min of heated and humidified oxygen, offers several physiological benefits over conventional oxygen therapy (COT). These include reduced anatomical dead space, maintenance of constant oxygen concentration, adequate humidification, and the provision of positive end-expiratory pressure [7]. At present, HFNC is considered an alternative to COT and noninvasive assisted ventilation (NIV) to prevent and treat postoperative acute respiratory failure in patients undergoing thoracic surgery, such as ESO, a procedure known for its high risk, and where ARISCAT scores may indicate significant risk even in the absence of other comorbidities [8–10]. This technique was also found to reduce the duration of hospital stay and the incidence of pulmonary infections after pulmonary and cardiac surgery [11–13].

Recently, specific data regarding the efficacy of HFNC in patients with hypoxemia following ESO have been relatively scarce. In this regard, the author retrospectively compared the arterial blood gas analysis of oxygenation index (PaO_2_/FiO_2_) before surgery, before oxygen therapy, and 6, 24, and 72 h after HFNC or COT in patients with ESO. Secondary outcomes encompassed partial pressure of carbon dioxide and blood oxygen saturation. Additionally, the study evaluated postoperative complications and assessed the viability of employing HFNC instead of COT for postoperative patients with EC.

## 2. Methods

### 2.1. Ethical Statement

Ethical approval (approval number: JD-HG-2022-25) was obtained from the Institutional Review Board of the Second Affiliated Hospital of Soochow University. The chairperson of the ethics committee in the Second Affiliated Hospital of Soochow University waived the need for patient consent due to the retrospective nature of our study.

### 2.2. Patient Selection

We conducted a retrospective review of the clinical records of 80 patients with hypoxemia who underwent ESO at the Second Affiliated Hospital of Soochow University between January 2020 and December 2022. These patients were subsequently divided into two groups: the HFNC group and the COT group.

### 2.3. Inclusion and Exclusion Criteria

Inclusion criteria were defined as follows: (1) diagnosis of EC was confirmed by CT, fiberoptic gastroscopy biopsy, and postoperative pathology and (2) patients with hypoxemia, which was defined as the ratio of arterial oxygen partial pressure to fractional inspired oxygen (P/F ratio) below 300 cmH_2_O, arterial or percutaneous oxygen saturation <  94% in room air, or partial pressure of arterial oxygen <  60 mmHg in room air or < 80 mmHg with oxygen [14].

Exclusion criteria were defined as follows: (1) those with an underlying clinical history of chronic obstructive pulmonary disease (COPD); (2) cardiogenic pulmonary edema; (3) patients underwent tracheotomy; (4) mental confusion, nausea, and vomiting; (5) impaired consciousness or disorientation; (6) hemodynamically unstable; (7) cardiac arrest; and (8) moderate or severe respiratory acidosis (pH < 7.30) combined with multiorgan failure [15].

### 2.4. Study Protocol

Patients in both groups underwent a similar protocol that included intravenous anesthesia with inhalational components, double-lumen endotracheal intubation, contralateral one-lung ventilation, thoracic access, and ESO. Patients who fulfilled the inclusion criteria were administered HFNC or COT oxygen therapy promptly following extubation. Concurrently, they received standard postoperative antibacterial therapy, including antibiotics targeted at specific pathogens identified in sputum cultures, as well as medications for acid suppression, hemostasis, and analgesia. Physiotherapy employs chest percussion, vibration, and postural drainage to facilitate the clearance of respiratory secretions. On this basis, the HFNC group was treated with Respircare's HUMID-BH high-flow humidified oxygen therapy system. The initial oxygen concentration was set at 50%, the flow rate at 40–50 L/min, and the temperature at 32°C–37°C to ensure continuous use and maintain saturation of blood oxygen (SaO_2_) above 95% [16]. The COT group was treated with a nasal cannula, and the flow rate was at 1–6 L/min. All subjects received postoperative oxygenation for at least 72 h.

### 2.5. Inspection Indicators

The primary indicators of the effectiveness of oxygen therapy were arterial blood gas analysis of oxygenation index (PaO_2_/FiO_2_) before surgery, before oxygen therapy, and 6, 24, and 72 h after oxygen therapy. The secondary indicator included partial pressure of carbon dioxide (PaCO_2_). The other indicators were partial pressure of arterial oxygen (PaO_2_) and SaO_2_ before surgery, before oxygen therapy, and 6, 24, and 72 h after oxygen therapy. Infection indicators were mainly C-reactive protein (CRP), calcitoninogen (PCT), white blood cell (WBC) count, and neutrophil (NEUT) count before surgery, before oxygen therapy, and 3 days after oxygen therapy. Additional indicators encompassed postoperative complications such as pneumonia, pleural effusion, atelectasis, esophagoenteric leak, mediastinitis, acute respiratory distress syndrome, and other postoperative complications defined by Esophagectomy Complications Consensus Group [17]. The duration of postoperative hospital stay was also recorded.

### 2.6. Statistical Analysis

Data were analyzed using IBM SPSS Version 27.0 (IBM Corp, Armonk, NY, USA.) statistical software. Continuous variables were expressed as mean ± SD or median (interquartile range [IQR]), and categorical variables were expressed as *n* (%). Data were compared between two groups using the *t*-test, *χ*^2^ test, or Fisher's exact test as appropriate. Repeated-measures ANOVA was used to estimate group effect, time effect, and interaction between group and time. In all analyses, a two-tailed *p* < 0.05 was considered statistically significant.

## 3. Results

### 3.1. Patients' Characteristics

**Figure 1:**
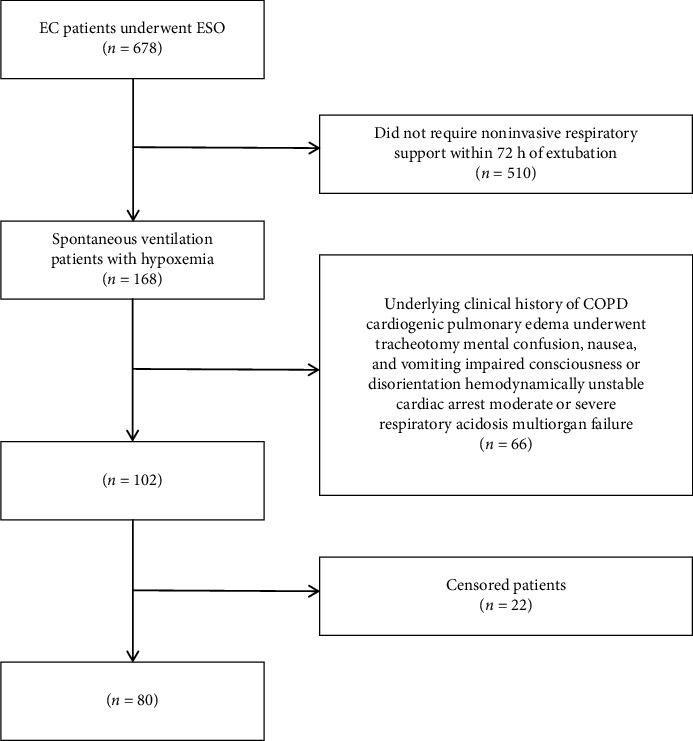
Consort flow diagram of the study.

Figure 1 shows the flow of participants through the trial. As shown in Table 1, the patients (50 males and 30 females) were aged 53–78 years, with a mean age of 66 years; their body mass index was 18.3–24.5, with a mean index of 21.6; the operation times ranged from 230 to 390 min (mean 312.8 min); there are 26 individuals underwent Ivor-Lewis ESO and 54 individuals underwent Mckeown ESO. There were no statistically significant differences between the groups in terms of gender, age, BMI, type of surgery, pulmonary function, and duration of surgery. The differences between the two groups were not statistically significant (*p* > 0.05) and were comparable.

### 3.2. Comparison of Patients' Arterial Blood Gases Between the HFNC Group and the COT Group

As shown in Table 2, the comparison of the interaction between group and time, time effect, and group effect of oxygenation index between the two groups of patients showed statistically significant differences (*P*_time∗group_ < 0.001, *P*_time_ < 0.001, *P*_group_ < 0.001). Analysis of individual effects revealed that the HFNC group demonstrated superior oxygenation indices compared to the COT group at 6 h, 24 h, and 72 h postinitiation of oxygen therapy (*P*_6h_ < 0.001,  *P*_24h_ < 0.001,  *P*_72h_ < 0.001). In addition, Table 3 shows that the comparison of PaCO_2_ between the two groups showed a statistically significant difference in the interaction between group and time, time effect, and group effect (*P*_time∗group_ = 0.027, *P*_time_ < 0.001, *P*_group_ < 0.001). After 6 and 24 h of oxygen therapy, there was a statistically significant difference in the decrease in PaCO_2_ in the HFNC group compared to the COT group (*P*_6h_ < 0.001,  *P*_24h_=0.010). Interestingly, the time effect on PaCO2 was statistically significant (*P*_time_ = 0.002), whereas the group effect and the interaction between group and time did not yield statistically significant differences (*P*_time∗group_ = 0.210, *P*_group_=0.746) (Table 4). A significant difference in respiratory rate was observed before and after surgery as well as following oxygen therapy; however, the use of HFNC did not have a significant impact on this parameter (*P*_time∗group_ = 0.350, *P*_time_ ≤ 0.001, *P*_group_=0.005) (Table 5). Furthermore, significant differences were found in blood oxygen saturation when analyzing the interaction between group and time, time effect, and group effect (*P*_time∗group_ = 0.517, *P*_time_ = 0.089, *P*_group_=0.172) (Table 6).

### 3.3. Comparison of Patients' Infection Indicators Between the HFNC Group and the COT Group

As shown in Table S1, the analysis of CRP levels revealed statistically significant differences in the interaction between group and time, as well as the time and group effects, across the two patient groups (*P*_time∗group_ < 0.001, *P*_time_ < 0.001, *P*_group_ < 0.001). After 3 days of oxygen therapy, there was a statistically significant difference in the reduction of CRP in the HFNC group compared with the COT group (*P*_3d_ < 0.001). Nevertheless, the difference was statistically significant when comparing the time effect of PCT (*P*_time_ < 0.001), yet the group effect and the interaction between group and time did not exhibit significant differences (*P*_time∗group_ = 0.064, *P*_group_=0.128) (Table S2). Similarly, there was a statistical difference in WBC count between the two groups with the time effect (*P*_time_ < 0.001), and comparison of group effect and interaction between group and time showed no statistically significant differences (*P*_time∗group_ = 0.156, *P*_group_ = 0.621) (Table S3). Nonetheless, comparison of the interaction between group and time, time effect, and group effect of NEUT count between the two groups of patients showed statistically significant differences (*P*_time∗group_ = 0.004, *P*_time_ < 0.001, *P*_group_ = 0.039). After 3 days of oxygen therapy, there was a statistically significant difference in the reduction of NEUT count in the HFNC group compared with the COT group (*P*_3d_ < 0.001) (Table S4).

### 3.4. Comparison of the Outcome Events Between the HFNC and COT Groups

After ESO in the two groups of EC patients, the length of hospital stay was shorter in the HFNC group than in the other group after different oxygen treatments (*p*=0.009). As shown in Table S5, the number of postoperative pneumonia cases was significantly lower in the HFNC group compared to the COT group (*p*=0.039), while the effect of different oxygen therapy on the occurrence of atelectasis (*p*=0.692) and pleural effusion (*p*=0.133) was not significant.

## 4. Discussion

Despite improvements in the perioperative management of EC, there is still a high incidence of postoperative complications after ESO, particularly postoperative pulmonary complications. In the study conducted by the authors, HFNC notably reduced the incidence of postoperative pneumonia, but the effect on atelectasis and pleural effusion was not significant compared to COT. Common postoperative complications include postoperative pneumonia and pulmonary atelectasis, which can occur in 13%–38% of postoperative EC patients [17–19]. HFNC has been reported to reduce the incidence of postoperative pulmonary complications and anastomotic fistula in EC compared to COT [15]. Nonetheless, a meta-analysis indicated that while HFNC lessened the incidence of reintubation and escalation of respiratory support relative to COT, it showed no statistical difference in the incidence of postoperative pulmonary complications and mortality compared with COT [20]. Additionally, a study which included 11 RCTs enrolling 2201 patients also suggests that the reduction in reintubation and escalation of respiratory support with prophylactic HFNC compared to COT is primarily observed in patients who are at high risk and/or obese [21]. Another meta-analysis suggested that HFNC could significantly reduce the length of hospital stay [22]. This finding is further corroborated by the results of this paper, potentially attributable to the management of postoperative pneumonia among patients.

COT cannot provide a stable oxygen concentration and humidified oxygen when administered through a nasal cannula, and its respiratory support is rather limited while it has a certain probability of causing sore throat [23]. In contrast, HFNC is increasingly being used because it can deliver up to 60 L/min of humidified oxygen continuously, is easy to use, improves patient tolerance, provides some positive end-expiratory pressure to improve alveolar recruitment, and enhances nasopharyngeal dead space flushing [24, 25]. It has been suggested that HFNC has various physiological effects, such as reduced inspiratory effort, enhanced lung volume and compliance, significantly increased oxygen saturation (SaO2%), and decreased respiratory rate. These benefits position HFNC as a viable alternative to COT for patients experiencing acute respiratory failure [26, 27]. In this study, the improvement in oxygenation index after 6, 24, and 72 h of oxygen therapy was significantly better in the HFNC group than in the COT group, suggesting that HFNC can improve the postoperative oxygenation of patients at an early stage and sustain it. After 6 h and 24 h of oxygen therapy, the HFNC group was more effective than the COT group in reducing PaCO_2_, but there was no significant difference between the two groups after 72 h of therapy, suggesting that HFNC can be used early in patients with hypoxemia with elevated PaCO_2_, but there is no significant advantage over COT for long-term use. In one study, HFNC was used in the early treatment of patients with severe COVID-19 and significantly improved CRP and NEUT count after 3 days of oxygen administration compared with COT. In the present study, HFNC improved CRP, PCT, and NEUT count better than conventional oxygen administration but did not have a statistically significant effect on WBC count [28]. HFNC is recommended for early-stage use in ESO patients to enhance infection indicators, aid in inflammation control, and decrease complications, potentially accelerating postoperative recovery.

We acknowledge there are some limitations in this study. First, it is a single-center study which should be reproduced in a multicenter one to improve its general applicability. Second, due to the fact that the middle-aged and elderly population is the high-risk group for EC, our research findings have limited generalisability among younger demographics.

## 5. Conclusions

In conclusion, HFNC improved their oxygenation index and PaCO2, and it improved infection indicators and decreased the risk of postoperative pneumonia in patients with hypoxemia after ESO.

## Figures and Tables

**Table 1:** Baseline characteristics of the included patient cohort (*N* = 80).

Characteristic	HFNC group (*n* = 40)	COT group (*n* = 40)	*p*
Sex			
Male	26 (65%)	24 (60%)	0.644
Female	14 (35%)	16 (40%)	0.517
Age (years)	66 ± 5	67 ± 6	0.454
BMI (kg/m^2^)	21.5 ± 1.8	21.8 ± 1.3	
Surgical procedure			
Ivor-Lewis	16 (40%)	10 (25%)	0.152
Mckeown	24 (60%)	30 (75%)	0.128
FEV1/FVC (%)	73.7 ± 4.9	75.5 ± 5.5	0.662
MVV (L/min)	93.5 ± 9.3	92.7 ± 6.8	0.489
Surgical time (min)	315 (235–410)	325 (285–400)	

Abbreviations: BMI, body mass index; COT, conventional oxygen therapy; FEV1, forced expiratory volume in one second; FVC, forced vital capacity; HFNC, high-flow nasal cannula; MVV, maximum ventilatory volume.

**Table 2:** Effect of different oxygen therapy modalities on oxygenation index (PaO_2_/FiO_2_) in 2 groups of patients ($\bar{x}\pm s$).

		Before surgery	Before oxygen therapy	6 h after oxygen therapy	24 h after oxygen therapy	72 h after oxygen therapy
HFNC group	*n* = 40	322.2 ± 10.62	264.55 ± 14.80	300.13 ± 17.19	327.35 ± 23.05	342.53 ± 24.74
COT group	*n* = 40	321.68 ± 12.13	265.53 ± 16.64	283.35 ± 22.28	300.60 ± 17.17	307.08 ± 16.22
*F*		*F*_time∗group_ = 19.752, *F*_time_ = 195.145, *F*_group_ = 41.779				
*p*		*P*_time∗group_ < 0.001, *P*_time_ < 0.001, *P*_group_ < 0.001				

Abbreviations: COT, conventional oxygen therapy; FiO_2,_ fraction of inspired oxygen; HFNC, high-flow nasal cannula; PaO_2,_ partial pressure of arterial oxygen.

**Table 3:** Effect of different oxygen therapy modalities on partial pressure of carbon dioxide in 2 groups of patients ($\bar{x}\pm s$).

		Before surgery	Before oxygen therapy	6 h after oxygen therapy	24 h after oxygen therapy	72 h after oxygen therapy
HFNC group	*n* = 40	40.37 ± 2.19	41.77 ± 1.58	39.83 ± 1.99	39.54 ± 2.28	39.60 ± 2.30
COT group	*n* = 40	40.46 ± 1.88	41.95 ± 1.43	41.67 ± 2.42	40.85 ± 2.17	40.29 ± 1.60
*F*		*F*_time∗group_ = 2.782, *F*_time_ = 11.030, *F*_group_ = 16.760				
*p*		*P*_time∗group_ = 0.027, *P*_time_ < 0.001, *P*_group_ < 0.001				

Abbreviations: COT, conventional oxygen therapy; HFNC, high-flow nasal cannula.

**Table 4:** Effect of different oxygen therapy modalities on partial pressure of oxygen in 2 groups of patients ($\bar{x}\pm s$).

		Before surgery	Before oxygen therapy	6 h after oxygen therapy	24 h after oxygen therapy	72 h after oxygen therapy
HFNC group	*n* = 40	89.26 ± 7.54	87.24 ± 5.35	89.76 ± 7.74	86.68 ± 5.34	90.53 ± 7.02
COT group	*n* = 40	91.28 ± 7.16	86.01 ± 3.65	88.60 ± 7.01	88.01 ± 5.74	88.53 ± 6.84
*F*		*F*_time∗group_ = 1.489, *F*_time_ = 4.514, *F*_group_ = 0.106				
*p*		*P*_time∗group_ = 0.210, *P*_time_ = 0.002, *P*_group_=0.746				

Abbreviations: COT, conventional oxygen therapy; HFNC, high-flow nasal cannula.

**Table 5:** Effect of different oxygen therapy modalities on respiratory rate in 2 groups of patients ($\bar{x}\pm s$**)**.

		Before surgery	Before oxygen therapy	6 h after oxygen therapy	24 h after oxygen therapy	72 h after oxygen therapy
HFNC group	*n* = 40	15.43 ± 2.41	15.30 ± 2.29	21.05 ± 1.83	19.68 ± 2.58	15.38 ± 2.43
COT group	*n* = 40	15.43 ± 2.30	15.58 ± 2.31	22.10 ± 1.41	20.15 ± 2.17	16.68 ± 2.70
*F*		*F*_time∗group_ = 1.111, *F*_time_ = 125.229, *F*_group_ = 8.271				
*p*		*P*_time∗group_ = 0.350, *P*_time_ ≤ 0.001, *P*_group_=0.005				

Abbreviations: COT, conventional oxygen therapy; HFNC, high-flow nasal cannula.

**Table 6:** Effect of different oxygen therapy modalities on saturation of blood oxygen in 2 groups of patients ($\bar{x}\pm s$).

		Before surgery	Before oxygen therapy	6 h after oxygen therapy	24 h after oxygen therapy	72 h after oxygen therapy
HFNC group	*n* = 40	97.47 ± 1.66	97.20 ± 1.68	97.58 ± 1.72	97.45 ± 1.75	97.28 ± 1.65
COT group	*n* = 40	97.25 ± 1.81	96.38 ± 1.89	97.35 ± 1.66	97.45 ± 1.80	97.35 ± 1.85
*F*		*F*_time∗group_ = 0.815, *F*_time_ = 2.038, *F*_group_ = 1.901				
*p*		*P*_time∗group_ = 0.517, *P*_time_ = 0.089, *P*_group_=0.172				

Abbreviations: COT, conventional oxygen therapy; HFNC, high-flow nasal cannula.

## Data Availability

Derived data supporting the findings of this study are available from the corresponding author on request.

## Supporting Information

Supporting Table 1: Effect of different oxygen therapy modalities on C-reactive protein in 2 groups of patients ($\bar{x}\pm s$).

Supporting Table 2: Effect of different oxygen therapy modalities on calcitoninogen in 2 groups of patients ($\bar{x}\pm s$).

Supporting Table 3: Effect of different oxygen therapy modalities on white blood cell count in 2 groups of patients ($\bar{x}\pm s$).

Supporting Table 4: Effect of different oxygen therapy modalities on neutrophil count in 2 groups of patients ($\bar{x}\pm s$).

Supporting Table 5: Effect of different oxygen therapy on postoperative length of stay and improvement of pulmonary complications in 2 groups of patients.
