# Supplementary material for: Effect of High-Flow Nasal Cannula Oxygen Therapy on Hypoxemia in Patients After Esophagectomy
Source: Can Respir J. 2025 Feb 20;2025:4691604. doi: 10.1155/carj/4691604 (PMC11867717; doi:10.1155/carj/4691604)
Supplement: Supporting Information — Additional supporting information can be found online in the Supporting Information section. [file 4691604.f1.docx]

**Supplementary** **Table1. Effect of different oxygen therapy modalities on C-reactive protein in 2 groups of patients (x̅±s)**

|  |  | Before  surgery | Before  oxygen therapy | 3d after  oxygen therapy |  |
| --- | --- | --- | --- | --- | --- |
| HFNC group | n=40 | 5.10±2.69 | 49.25±17.55 | 18.18±6.84 |  |
| COT group | n=40 | 4.66±2.50 | 54.02±15.35 | 35.56±11.62 |  |
| *F* |  | *F*_time*group_=17.563, *F*_time_=458.581, *F*_group_=17.603 | | | |
| *P* |  | *P*_time*group_<0.001, *P*_time_ <0.001, *P*_group_<0.001 | | | |

Abbreviations: HFNC, High-Flow Nasal Cannula; COT, Conventional oxygen therapy.

**Supplementary** **Table2. Effect of different oxygen therapy modalities on calcitoninogen in 2 groups of patients (x̅±s)**

|  |  | Before  surgery | Before  oxygen therapy | 3d after  oxygen therapy |  |
| --- | --- | --- | --- | --- | --- |
| HFNC group | n=40 | 0.03±0.01 | 0.66±0.23 | 0.28±0.13 |  |
| COT group | n=40 | 0.03±0.02 | 0.68±0.21 | 0.37±0.14 |  |
| *F* |  | *F*_time*group_=2.991, *F*_time_=514.055, *F*_group_=2.361 | | | |
| *P* |  | *P*_time*group_=0.064, *P*_time_ <0.001, *P*_group_=0.128 | | | |

Abbreviations: HFNC, High-Flow Nasal Cannula; COT, Conventional oxygen therapy.

**Supplementary** **Table3. Effect of different oxygen therapy modalities on white blood cell count in 2 groups of patients (x̅±s)**

|  |  | Before  surgery | Before  oxygen therapy | 3d after  oxygen therapy |  |
| --- | --- | --- | --- | --- | --- |
| HFNC group | n=40 | 7.08±1.53 | 11.13±0.89 | 10.03±0.95 |  |
| COT group | n=40 | 6.62±1.51 | 11.41±0.99 | 9.99±1.08 |  |
| *F* |  | *F*_time*group_=1.909, *F*_time_=283.413, *F*_group_=0.246 | | | |
| *P* |  | *P*_time*group_=0.156, *P*_time_ <0.001, *P*_group_=0.621 | | | |

Abbreviations: HFNC, High-Flow Nasal Cannula; COT, Conventional oxygen therapy.

**Supplementary** **Table4. Effect of different oxygen therapy modalities on neutrophil count in 2 groups of patients (x̅±s)**

|  |  | Before  surgery | Before  oxygen therapy | 3d after  oxygen therapy |  |
| --- | --- | --- | --- | --- | --- |
| HFNC group | n=40 | 3.51±0.79 | 7.67±0.80 | 5.48±0.64 |  |
| COT group | n=40 | 3.25±0.78 | 7.94±0.65 | 6.00±0.64 |  |
| *F* |  | *F*_time*group_=5.615, *F*_time_=695.409, *F*_group_=4.395 | | | |
| *P* |  | *P*_time*group_=0.004, *P*_time_ <0.001, *P*_group_=0.039 | | | |

Abbreviations: HFNC, High-Flow Nasal Cannula; COT, Conventional oxygen therapy.

**Supplementary** **Table5. Effect of different oxygen therapy on postoperative length of stay and improvement of pulmonary complications in 2 groups of patients**

| Group | Cases | Median time in hospital  after surgery (d，**x̅±s**) | Postoperative pulmonary complications (n,%) | | | |
| --- | --- | --- | --- | --- | --- | --- |
|  |  |  | Pulmonary infections | Pulmonary atelectasis | Pleural effusion | |
| HFNC | 40 | 16.8±6.1 | 6(15%) | 3(7.5%) | | 10(25%) |
| COT | 40 | 20.4±5.8 | 14(35%) | 4(10%) | | 18(45%) |
| *t*/χ^2^ |  | -2.69 | 4.267 | 0.157 | | 2.257 |
| *P* |  | 0.009 | 0.039 | 0.692 | | 0.133 |

Abbreviations: HFNC, High-Flow Nasal Cannula; COT, Conventional oxygen therapy.
